# From signals to music: a bottom-up approach to the structure of neuronal activity

**DOI:** 10.3389/fnsys.2023.1171984

**Published:** 2023-08-11

**Authors:** Gabriel D. Noel, Lionel E. Mugno, Daniela S. Andres

**Affiliations:** ^1^College of Interdisciplinary and Advanced Studies in the Social Sciences, National University of San Martin (UNSAM), San Martín, Argentina; ^2^National Scientific and Research Council, National University of San Martin (UNSAM), Buenos Aires, Argentina; ^3^School of Music of the Department of General San Martin “Alfredo Luis Schiuma”, San Martín, Argentina; ^4^Institute of Emergent Technologies and Applied Science, San Martín, Argentina; ^5^Science and Technology School, National University of San Martin (UNSAM), San Martín, Argentina

**Keywords:** structuralism, linguistics, Lévi-Strauss, music, structural hearing, neural code, fractals

## Abstract

**Introduction:**

The search for the “neural code” has been a fundamental quest in neuroscience, concerned with the way neurons and neuronal systems process and transmit information. However, the term “code” has been mostly used as a metaphor, seldom acknowledging the formal definitions introduced by information theory, and the contributions of linguistics and semiotics not at all. The heuristic potential of the latter was suggested by structuralism, which turned the methods and findings of linguistics to other fields of knowledge. For the study of complex communication systems, such as human language and music, the necessity of an approach that considers multilayered, nested, structured organization of symbols becomes evident. We work under the hypothesis that the neural code might be as complex as these human-made codes. To test this, we propose a bottom-up approach, constructing a symbolic logic in order to translate neuronal signals into music scores.

**Methods:**

We recorded single cells’ activity from the rat’s globus pallidus pars interna under conditions of full alertness, blindfoldedness and environmental silence. We analyzed the signals with statistical, spectral, and complex methods, including Fast Fourier Transform, Hurst exponent and recurrence plot analysis.

**Results:**

The results indicated complex behavior and recurrence graphs consistent with fractality, and a Hurst exponent >0.5, evidencing temporal persistence. On the whole, these features point toward a complex behavior of the time series analyzed, also present in classical music, which upholds the hypothesis of structural similarities between music and neuronal activity. Furthermore, through our experiment we performed a comparison between music and raw neuronal activity. Our results point to the same conclusion, showing the structures of music and neuronal activity to be homologous. The scores were not only spontaneously tonal, but they exhibited structure and features normally present in human-made musical creations.

**Discussion:**

The hypothesis of a structural homology between the neural code and the code of music holds, suggesting that some of the insights introduced by linguistic and semiotic theory might be a useful methodological resource to go beyond the limits set by metaphoric notions of “code.”

## 1. Introduction

From at least the beginning of the twentieth century, language has been moving to the forefront of research on both the sciences and the humanities. Furthered by parallel advances in analytical philosophy and the sciences of language, this “linguistic turn” gave birth to research programs in many disciplines, from literary theory to biology, from sociology and anthropology to architecture, from engineering to music, especially during the apex of the intellectual movement called “structuralism,” between the end of the fifties and the beginning of the seventies (Rorty, 1979; Eagleton, 1983; Dosse, 1998). The structural approach aimed to apply formal procedures to empirical data in order to turn them into logico-mathematical models that might be expressed/derived through series of transformations. Its research agenda proposed that language - or rather the formal models of language built by structural linguistics and phonology - could be used as a basis to build homologous models of empirical phenomena potentially involving any kind of communication^1^ (Jakobson, 1962; Trubetzkoy, 1969; de Saussure, 2011). Communication, in turn, implies the notion of “code,” defined as “a set of symbols and the rules for their combination, which can be used to represent a pattern of information” (Uttal, 1969; Eco, 1978). However appealing, the idea of a straightforward, single-level code as a sufficient representation of communication processes is too simplistic for most applications and has been progressively discarded and replaced with the concept of multiplexed codes, particularly in neuroscience (Andres, 2015). The necessity of such an approach becomes evident in the analysis of the most elaborate of codes produced by human beings: language and music. Both constitute complex communication systems, which exhibit a multilevel and layered structure that brings about interrelated effects at the cognitive, affective and intersubjective levels.^2^ Approaches and tools suitable for the analysis of these rich and nuanced codes have been proposed both on the side of social sciences - by linguistics, which in turn gave birth to structuralism - and mathematics - with the development of fractal formalisms. As early as 1975, Voss and Clarke (1975) proposed fractal characteristics as defining traits in the creation of algorithmic music, and fractal properties have been widely studied both in musical pieces (in particular in the classical genre) and in neuronal signals (Trulla et al., 2018; Rolla et al., 2021).

On the other hand, the term “neural code” has been frequently applied in neuroscience to the process of coding and decoding of information by neurons, as well as the transmission of this information through neural networks (Gerstner et al., 1997; Naci et al., 2014; Andres et al., 2015). However, in many cases, approaches to “code” follow the metaphors that happen to be current (Turner, 1975). A poignant critique warning about the pitfalls of a metaphoric approach to cognition and neuroscience can be found in a recent article by Cobb (2021). The arguments are particularly appropriate to the casual treatments of “code” made frequently in neuroscience, which seldom acknowledge the specific conceptual treatment introduced by linguistics and semiotics several decades ago to deal with matters such as the distinction between signal and symbol, the difference between information and signification or the problems related to the relationships between syntax, semantics and behavior (Eco, 1978). Historically, the neuroscientific approach has tied meaning to context, in terms of standardized experimental settings mainly designed to deal with sensory systems. Classic research in the field was conducted by Uttal, one of the pioneers in defining and applying the idea of code to sensory neural systems (Uttal, 1969). As much as this foundational work set the basis for current views on the sensorimotor system and led to a thriving field of applications on neural control and brain-machine interfaces, a notion of code consistent with linguistic and semiotic developments should move forward the analysis of signification as a property intrinsic to the multilayered structure of codes themselves. Something similar happens with concepts such as entropy and information applied to the problem of communication between neurons. Even when they have allowed for the quantitative study of neural processes, they run the risk of impoverishing the analysis, since they may not account for the different superimposed systems that can be deployed to produce meaning.

We work under the hypothesis that the neural code shares some properties with the most complex and multilayered encoding systems of human making: music and language. To test this hypothesis, we conduct an experiment that reverts the process undertaken by original structuralist analysis. Instead of going downwards in search of the human mind by the way of its most complex productions, we build a bottom-up approach, starting from the lowest level of abstraction - i.e., neuronal signals - and constructing a symbolic logic in order to translate those signals into music scores (Church, 1936). The experiment allows for a meaningful comparison between music and raw neuronal activity which suggests, when subjected to mathematical analysis, that their structures are in fact homologous. This lends credence to the original insights of the structuralist perspective from a novel point of view and opens up the possibility of using a linguistic approach for the analysis of the neural code.

## 2. Materials and methods

### 2.1. Experimental procedure

The neuronal recordings used for the present work were originally produced within an experimental Parkinson’s research protocol that followed the partial-lesion model originally described by Sauer and Oertel (1994). The signals analyzed correspond to microelectrode recordings (MER) obtained from adult rats weighing 250–350 g. assigned to the control group of the aforementioned protocol (Andres et al., 2014). The goal of the experiments was to record the spontaneous activity of the globus pallidus pars interna (GPi, previously called entopeduncular nucleus) under conditions of alertness, environmental silence and blindfoldedness.^3^ Optimal anesthetic and analgesic medication was used for the recording surgeries, with a three-drug profile consisting of a combination of tramadol, lidocaine and chloral hydrate. Following anesthesia, animals were placed in a restraining device built *ad hoc* with semi-rigid plastic and a high-quality thermal insulator on the inside. Once the animals were placed, fixed to the stereotactic frame and ready for surgery, we waited until they were completely awake, given the objective was to record spontaneous neuronal activity under complete alertness. The awakening process was monitored periodically with a standardized evaluation of the tail reflex. The purpose of the device was to minimize the animals’ discomfort during the surgery. During the whole procedure animals remained only loosely bound. If they didn’t relax but attempted to move, that was considered an endpoint and the recording surgery was terminated. During the whole surgical procedure, the eyes of the animals were covered and all surgeries were conducted in identical conditions of environmental silence. Recording coordinates fell within the limits defined as the GPi by the last edition of Paxinos and Watson’s (1997) Atlas. Neuronal recordings were obtained using glass-insulated platinum/iridium (Pt/Ir 80/20%) microelectrodes with nominal impedance of 0.8 – 1.2 megohms (mTSPBN-LX1, FHC Inc, Bowdoin, ME, USA). Signals were amplified, conditioned and monitored with an analog oscilloscope, digitized with a dedicated acquisition system (1401 plus, CED) and saved in a computer running Spike 2.0 software. The sampling rate was 20 kHz and total amplification including probe was × 10,000, checked with a built-in calibration signal of 1 mV p-p at the beginning of each experiment.

### 2.2. Signal analysis

Signals were processed offline. Spikes were extracted and classified using the algorithm developed by Quian Quiroga et al. (2004). Single units were used to construct time series of interspike intervals (ISI) in the following way:


$$X=\left\{\left(x_{i+1}-x_{i}\right),\ldots ,\left(x_{n}-x_{n-1}\right)\right\},$$

with *x_i_* corresponding to the times of occurrences of successive spikes. Thirty seconds of recording following the application of the tail-stimulus were discarded from each time series in order to warrant stationarity. Time series were used for statistical, spectral and non-linear analysis as described below. All calculations were performed with custom code run on Matlab^®^.

#### 2.2.1. Statistical analysis

Basic descriptive statistics were calculated for every time series: mean, standard deviation, mode, median, percentiles, skewness and kurtosis. The Anderson–Darling test was used to check whether neuronal activity was normally distributed. Histograms were adjusted to various models to find the best fit of the probability distribution of neuronal activity. All results are presented as mean ± standard error of the mean (SEM).

#### 2.2.2. Spectral analysis

The spectral properties of the time series were analyzed with a Fast Fourier Transform (FFT). For each time series, a running average with a 30-point window was applied to smoothen the signal, shortening it 30 points on the right end. Subsequently, the amplitude of the FFT was plotted on a double logarithmic graph. Since power spectra of systems with a complex behavior obey a power law like the following:


$$P\,\left(\omega \right)\propto \omega^{-\beta },$$

the double logarithmic plot can be used to recover the spectral exponent β as the slope of the linear regression to the spectrum. Linear regressions were only accepted when the regression coefficient *R*^2^ ≥ 0.8.

#### 2.2.3. Hurst exponent

We calculated the Hurst exponent based on the classic method of rescaled range analysis. This exponent captures the long-term memory effects present in time series and can be extracted from the following relation:


$$\frac{R}{S}\propto \tau^{H},$$

where *R* is the signal range, *S* the standard deviation, τ the temporal scale and *H* the Hurst exponent. Plotting this relation in double logarithmic axes, the exponent *H* is obtained as the slope from the linear regression. Linear regressions with *R*^2^ < 0.8 were discarded from the present study. Interestingly, a formal relation is verified between the Hurst and the spectral exponents, namely *H* and β: for processes that can be modeled by a fractional Brownian motion (fBm) and when 0 < β < −1, the exponent $H=\frac{\beta +1}{2}$ (Mandelbrot, 1985; Wu et al., 2015). This allows for a direct comparison between results obtained with rescaled range and spectral analysis. We performed this comparison and expressed the results as a percentage for the difference of *H* obtained by both methods.

#### 2.2.4. Recurrence plots

The unthresholded recurrence plot was calculated applying the method published by Yang (2011). A color scheme was used to represent the value of the norm between ISI *x_i_* and *x*_*i+τ*_ separated by a time distance (in indexed time units) of τ. Plots were calculated for three different data lengths (*n* = {10^2^, 10^3^, 10^4^}) in order to look for self-affine properties.

### 2.3. Music composition

**FIGURE 1:**
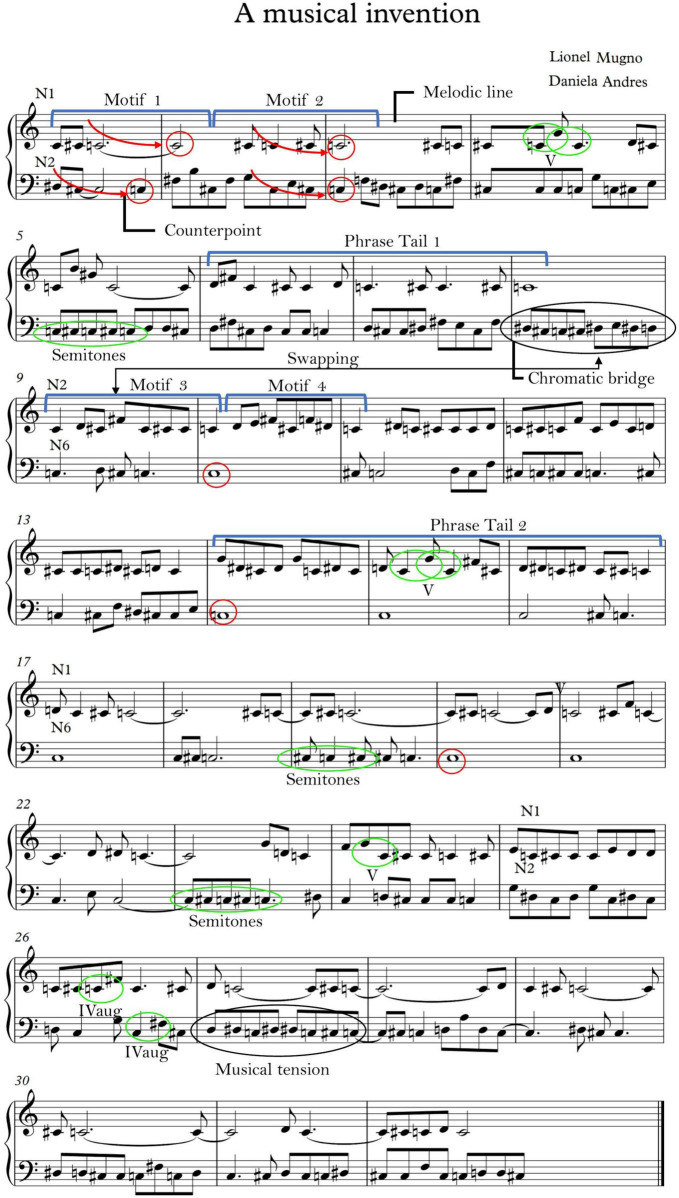
Musical score produced by transcribing neuronal signals using a symbolic logic and its compositional analysis. Randomly selected segments of neuronal signals were used for this construction (labeled *N*_1_, *N*_2_, *N*_6_ in the music sheet). Note how several musical elements appear recurrently in segments produced from different neurons (e.g., bar 14 on treble key and 25 in bass key). The leit motiv of the piece is C - C# - C, with a constant direction toward C, which works as the piece’s fulcrum. From a pure stylistic point of view the finale on C# may be interpreted as an unexpected finale, redolent of a modulation at the last bar to the ionian mode, typical of Bach (e.g., Bach Nr. 2 BWV 847 c-Moll).

A symbolic logic was created in order to translate the recorded signals into music scores. Starting from the neuronal time series, a coarse graining was applied, discretizing the series range in 12 intervals of equal amplitude, and semitones ranging from C to B were assigned to each of the successive segments. To achieve meaningful results from a musical standpoint, notes’ duration was set to quaver as the default value, except for repeated intervals of the smallest value, in which case the number of successive ISI was added to account for a duration of breve, semibreve, minim, and so on. The musical piece presented here was built with signals obtained from three neurons (segments labeled *N*_1_, *N*_2_, *N*_6_, Figure 1), alternatively used as melodic line and counterpoint to produce musical texture. Signal segments were selected randomly from MER recordings. We then performed a compositional analysis following the principles of structural hearing set by Salzer (1982). These principles state that harmonic progressions (typically expressed as I - IV - V intervals) are present in semiphrases, phrases, sections or whole movements of a musical piece, i.e., harmony is expressed at different scales and in nested structures, akin to self-affine properties. We applied this analysis to the sheet music produced, looking for harmonic progressions, motives, tension and resolution, and most importantly, patterns that might resemble human-made music.

## 3. Results

**FIGURE 2:**
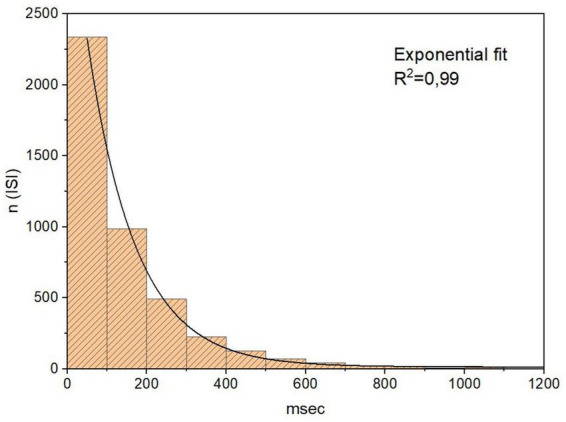
Histogram of a sample time series of neuronal activity and corresponding fitting. As for every case studied, the distribution is positively skewed (asymmetric to the left) and non-gaussian. The logistic ($y=\frac{A_{1}+A_{2}}{1+{\left(x/x_{0}\right)}^{p}}+A_{2}$), poissonian ($y=y_{0}+\frac{e^{-r}\cdot r^{x}}{x!}$) and exponential (*y* = *y*_0_ + *Ae*^*R*_0_*x*^) functions were tried to fit the distribution; the best fit corresponded to an exponential function of the form *y* = 14,31 + 3483, 86⋅*e*^−0,008⋅*x*^.

**FIGURE 3:**
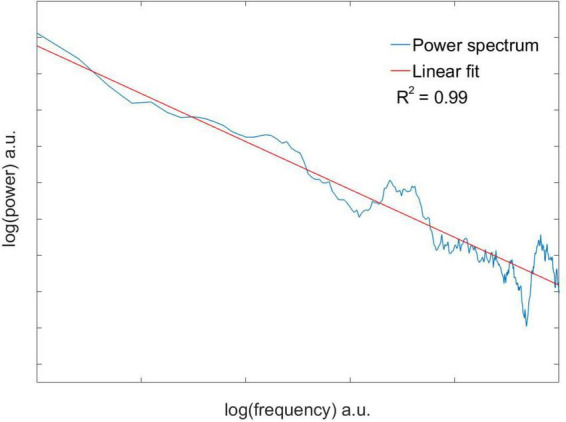
Double logarithm of the power spectrum with the corresponding linear fit of a sample time series of neuronal activity. An exponent −β=0.11 was recovered, indicating a complex behavior. Since time series are expressed in numbered intervals, the power spectrum exhibits arbitrary units (a.u.).

**FIGURE 4:**
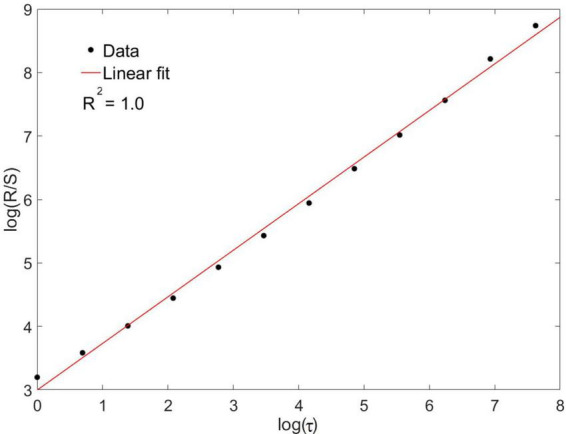
A rescaled range analysis was applied for the calculation of the Hurst exponent; the figure shows the implementation for a sample time series. The exponent was recovered as the slope of the linear regression fit to the relation between *R*/*S* and τ in double logarithmic axes, where *R* is the signal range, *S* the standard deviation and τ the temporal scale. For this sample case *H* = 0.73.

**FIGURE 5:**
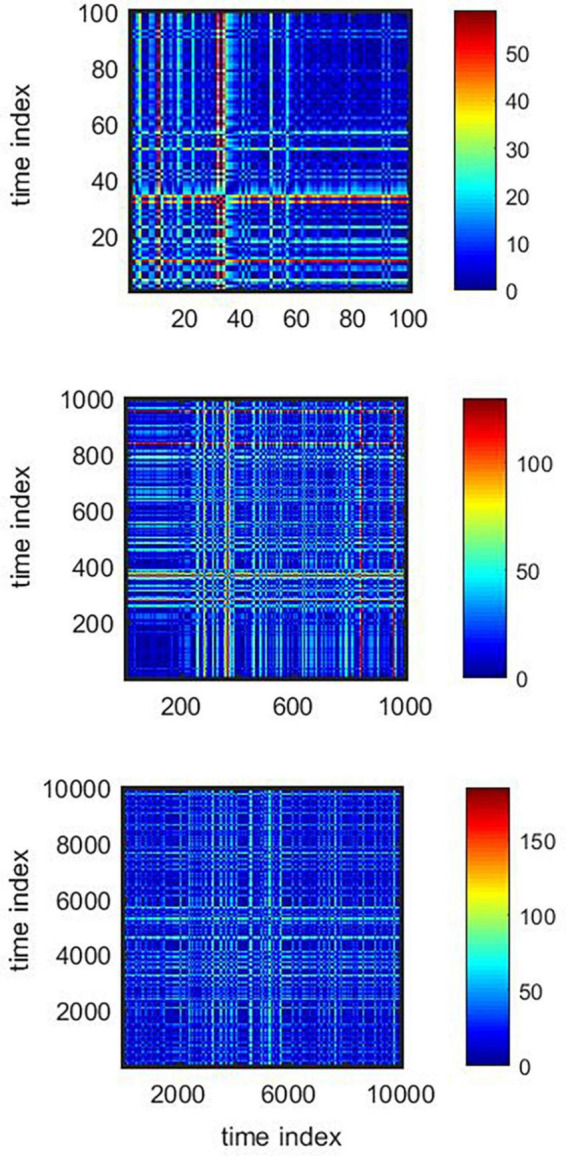
Colored recurrence plots of a sample neuronal signal for increasing data sizes (*n* = 10^2^, 10^3^, 10^4^). The self-affine properties of the time series are evident from the qualitatively similar behavior of the plots at increasing lengths.

Eleven neuronal recordings were obtained under the conditions outlined above. Descriptive statistics are presented in Table 1. As can be expected for neuronal activity of the GPi, the histograms of the time series were positively skewed, i.e., asymmetric to the left. The Anderson–Darling test rejected the null hypothesis of a gaussian distribution for every signal with a significance level higher than 0.05. Logistic ($y=\frac{A_{1}+A_{2}}{1+{\left(x/x_{0}\right)}^{p}}+A_{2}$), poissonian ($y=y_{0}+\frac{e^{-r}\cdot r^{x}}{x!}$) and exponential (*y* = *y*_0_ + *Ae*^*R*_0_*x*^) functions were tried to fit the histograms. Among the functions tried, the only one able to fit every case was the exponential function, with an *R*^2^ ≥ 0.8 in 9 out of 11 cases. Figure 2 shows the histogram of a sample time series of neuronal activity and the corresponding fit. From the power spectra an exponent −β ≥ 0.05 ± 0.02 was recovered, exhibiting the complex behavior of the time series, with *R*^2^ ≥ 0.8 for every case (Figure 3). The Hurst exponent exhibited a value of *H* > 0.5 (*H* = 0.7 ± 0.02) also for every case, showing that the neurons are persistent in terms of their long-term behavior (Figure 4). Recurrence plots displayed the typical behavior of complex systems, with a grid dominated mainly by vertical and horizontal lines and without strong diagonals, in a multicolored scheme related to the long-term correlations present in the signals (Figure 5). Furthermore, recurrence plots built at scales of increasing size (10^2^, 10^3^, 10^4^ data) were qualitatively similar, lending further evidence to the fractal (self-affine) nature of the neuronal activity studied, in addition to the behavior of both β and *H* exponents. The calculation of *H* with the spectral method (i.e., applying the aforementioned relation to the value of β) elicited a value of *H* = 0.52 ± 0.16, still within the temporal persistence range, with a difference of 18% between methods.

**TABLE 1:** Statistical properties of the time series analyzed.

Time series length	16887 ± 5548 ISI
Mean	130.89 ± 29.59 ms
Standard deviation	179.07 ± 53.02 ms
Skewness	2.21 ± 0.23 ms^3^
Kurtosis	8.23 ± 5.25 ms^4^
Mode	14.92 ± 7.48 ms
Minimum	1.05 ± 0.67 ms
Maximum	1626.39 ± 453.82 ms
Median	61.99 ± 15.70 ms
Percentile 25	15.63 ± 5.76 ms
Percentile 75	175.38 ± 43.55 ms

Results are presented as mean ± standard error of the mean.

Segments of MER from three neurons were used to compose a musical piece following the procedure described previously; an average of 182 intervals were used from each signal (*N*_1_ : 247 *ISI*, *N*_2_ : 168 *ISI*, *N*_6_ : 130 *ISI*). A compositional analysis of the piece, from a structural audition standpoint, elicited the results presented in Figure 1 (Salzer, 1982). The process of translating neuronal signals to musical notation produced a repertoire of set melodies. The samples selected from such repertoire to work as the main melodic line and as their counterpoint, respectively, established what we may call an interneuronal musical dialogue. Through this dialogue, neuronal utterances became related in a meaningful fashion, following a melodic line articulated as a mosaic of preexisting tunes. From design, and according to standard procedures of musical composition, the piece is divided in an eight-bar phrase structure. The score resulting from neuronal activity can be considered, from a musical point of view, an invention. Technically, this refers to a short composition in two-part counterpoint resembling a fugue, although simpler in structure (Bas, 1990).

Although the neuronal signals used as a basis for the musical score were selected randomly, the criteria guiding the creative process was the musical principle of “filling up space,” a compositional resource based on compensation of long-duration musical notes by means of shorter notes, in a search for continuity of melodic discourse in a counterpoint of two or more voices. In this way, since the melody taken as the starting point in bars 1–8 has a minimalist quality (*N_1_*), the one chosen as its counterpoint (*N_2_*) was selected according to its potential for complementing the melodic and rhythmic patterns of the first. Interestingly enough, even when we did not set out explicitly to build transitions or bridges between musical segments, they appeared spontaneously, as a result of the very structure of the piece and the nature of the signals themselves. For an example, note the bridge between bars 8 and 9, where we find a chromatic descent transition to the C in the latter. After this, a swapping takes place, in which *N_2_* moves from treble clef to bass clef. Throughout the piece, the note C works as a harmonic attraction point, a fulcrum, drawing the melody to itself in an oscillation of movements deployed in recurring motifs and phrases (e.g., in bars 1–3 in treble clef and bass clef, and also in bass clef in bars 10–20, among others). A harmonic analysis shows the semitone (especially the one between C and C#) to be a recurring feature of the score (n.b. the chromatic bridge in bar 8). The sequence C - C# - C appears time and again, which constitutes a stylistic element called ascending chromatic ornamentation. Other prominent intervals can be found, as the major fifth intervals in bars 4, 15 and 24, which also appears elsewhere in the piece, or the augmented fourth interval (e.g., bars 26 and 32). These recurring patterns found in different neurons amount to a resemblance of style (e.g., bars 4 and 15 in treble clef, or 5, 8 and 27 in the bass clef, an expression that builds musical tension). Motifs even become embellished and elaborated: the musical motifs presented in bars 1 and 2, for instance, are elaborated in bars 3–5, ending with a phrase tail in bars 6–9 (treble clef). Analogously, the motif in bars 9–10 are further elaborated in bars 11 and 13 concluding in a tail in bars 14–16 (treble clef). Interestingly, the same motifs and sequences are present in different signals (originated by different neurons) evidencing what can be construed as a dialogue between them. Taken together, these features, if found on the work of a human composer, would point to a certain stylistic coherence, a compositional grammar or authorial mark susceptible to further development in terms of an *oeuvre*. Note in this respect the tail-end of the musical phrase in bars 6 and 7, which works as a sort of coda leading to a satisfactory resolution.

## 4. Discussion

As we have stated previously, we set out to construct an experiment allowing for a comparison between music and raw neuronal activity. Through a bottom-up approach, starting from neuronal signals and constructing a symbolic logic in order to translate them into music scores, we show that their structures are homologous, that is they share basic structural properties. A lot of work has been devoted to the mathematical structure of music, dealing with different genres even when most of the literature has focused on classical music (Wu et al., 2015). In general terms, scale-free and fractal properties have been studied across styles and composers. Two features in particular have been covered more or less extensively, namely the spectral (β) and Hurst (*H*) exponents, both of which describe the complex properties of a system and are related to fractal geometry and self-affinity. The spectral exponent characterizes power spectra that decay following a power law 1/β, typical of systems with complex behavior. This kind of behavior has been found in neural systems at different scales (neuronal, microcircuitry and brain levels), and is also a typical feature of classical musical pieces (Hsü and Hsü, 1990, 1991; Bedard et al., 2006; Liu et al., 2013; Plenz et al., 2021). In fact, in their foundational paper, Voss and Clarke (1975) went as far as proposing that a 1/*f* power spectrum should be the defining trait in the creation of algorithmic music. Furthermore, different authors have argued that 1/*f* noise is a required feature of pleasant music (Teixeira Borges et al., 2019). This kind of spectral decay has been found in compositions by Bach, Brahms, Mozart and Beethoven; the Hurst exponent, in turn, has been calculated for Bach, Mozart, Palestrina, Haydn, Dvorak and Shostakovich, among others (Wu et al., 2015; González-Espinosa et al., 2017). Regarding the latter, introduced by Hurst (1956) in his cardinal paper, it is known to capture the long-term memory effects present in time series, allowing for classification of complex systems according to their persistent/antipersistent behavior (H > 0.5 and H < 0.5, respectively). It should be noted that the Hurst and spectral exponents are formally related, allowing for a mutual transformation. That is, while *H* is usually calculated with the rescaled range analysis (employed by us) or the detrended fluctuation analysis methods, it can also be obtained through spectral analysis. In practice, the simultaneous calculation of β and *H* provides a way of corroborating the robustness of the results and of finding bounds to the value of *H*. In the present work, we obtained values of *H* > 0.5 through both procedures, indicating persistent temporal properties of the neuronal time series. This is coincident with observations of classical music, in which *H* also exhibits persistence in a vast majority of cases (González-Espinosa et al., 2017). Taken together, these observations point toward mathematical similarities between neuronal activity and music, and also highlight the role of complex and scale-free properties in both domains.

Scale invariance and fractal properties in musical pieces have been found using other methods as well. In a recent work, Rolla et al. (2021) provide an in-depth analysis of fractality of 21 musical pieces from various composers, including Chopin, Haydn, Clementi, Mozart, Schubert, Brahms and Beethoven. Applying a network analysis, the authors emphasize that scale-free properties of music are present in the harmonic domain as much as in pitch and rhythm. Another work on pieces by Bach, Mozart and Beethoven showed that recurrence plots evidence a form of determinism and propose that determinism shows a progression toward the end of the pieces (Fukino et al., 2016). Trulla et al. (2018) also analyzed musical consonance and dissonance using recurrence plots and the notion of self-similarity. Although recurrence plots are not an ideal tool for quantitative analysis, they provide a friendly visual representation of time series at various scales. We calculated unthresholded recurrence plots of neuronal activity for increasing data lengths (10^2^, 10^3^, 10^4^), obtaining similar qualitative features for all the scales studied. These results support the hypothesis of local and global similarities in the structure of the studied signals (self-affinity) and are once again consistent with the observations in classical music.

For a thorough assessment of the present work, some technical points need to be addressed regarding the validity of interpretations built on extremely small data sets or short time series, as well as the relationship between the results, the translation criteria and the symbolic logic employed. Regarding sample size and time series length, too often the necessary conditions for the implementation of a method are overlooked, paradoxically leading to excessive demands on the side of data quality, based on the premise that overly long, regular, and abundant data would eventually allow for the implementation of any method. However, real data present effective challenges and constraints. Studying statistical, spectral, and non-linear properties of neuronal activity, we show that these properties are consistent with the presence of fractality and self-affinity in neuronal recordings of the rat’s GPi. All the numerical methods applied in our work are vulnerable to the number of data analyzed, with results depending on the size of data sets. This is compounded when looking for self-affine properties, since multiple scales need to be analyzed, making the data size problem even more critical. An approach that may allow for conclusions to be drawn from sets in the order of 10^2^ data, capable of providing new insights into a systems’ behavior, would be unfathomable in the field of time series analysis. Working across disciplines and applying a novel method, we were able to present a rigorous analysis of a neuronal signal by the way of structural hearing, with as few as 100–200 data points. The fact that patterns of neuronal activity (a central theme on neuroscientific endeavors) were found by means of compositional analysis, compared between different neurons and interpreted as a language is both surprising and revealing. This points to the potential heuristic fecundity of a theoretical and methodological dialogue between linguistics, semiotics, mathematics and neuroscience.

A second technical issue that needs to be addressed involves the possibility that the results presented might be a reflection of the translation method and the symbolic logic chosen. To a certain degree this can be considered to be the case, since the goal of our experiment was to show that such a translation is indeed possible. This should by no means be taken as a suggestion that our method is the only valid one, or that its results constitute the only valid solution to the problem presented. On the contrary, the idea of our work is to show a homology between the communication system used by neurons and complex human communication codes, which enables the translation between the former and the latter. Other attempts have been made on the creation of music from brain signals, the analysis of said signals in terms of musical notation or the resource to music as illuminating analogy. Baslow (2009), for instance, in his analysis of neuronal communication in terms of a spike/pause “language,” translates signals in terms of electronically generated musical notes based on frequency equivalents. Other researchers have dealt with the commonalities between the processing of language and music or the neural correlates of music performance or appreciation (Globerson and Nelken, 2013; Sutherland et al., 2013; Tierney and Kraus, 2013; Chiang et al., 2017). Our own approach, in turn, is marked by a homologic use of both compositional theory and structural hearing, harking from musical theory, and made possible by wide and far-reaching interdisciplinary cooperation.

This work echoes a long-standing program from the social sciences known as “structuralism.” Inspired by the early advances in formalization achieved by structural linguistics (and phonology in particular), structuralists, originally in France but later in other latitudes as well, set to apply the methods and findings of this discipline to other fields of knowledge, with the intention of constructing rigorous relational models susceptible to mathematical and/or logical treatment (Jakobson, 1962; Trubetzkoy, 1969; de Saussure, 2011). Claude Lévi-Strauss (1908–2009), the most ambitious of its proponents strived for an overarching science of the systems of meaning/signification (i.e., “codes”) called “semiotics” that would translate and inscribe models built for specific dominions into homologous and more abstract “structures” susceptible to an axiomatic formulation and deductive procedures of inference (Eco, 1978). The main assumptions of Lévi-Strauss’ perspective are dependent on his claim that linguistics, a human science, has achieved a formal status (and therefore a rigor) comparable to “natural” or “physical” sciences, leading, pointing and paving the way for other endeavors from the human (or social) domain, whose objects may be subjected to analogous treatment. In his case, as an anthropologist, these subject matters featured, prominently, kinship systems, social organization and classification systems and mythology (Lévi-Strauss, 1955, 1969, 1971, 1983a,b,c,d, 1988a,b, 1996, 2021). It must be stressed that, unlike many researchers aligned with the aforementioned “linguistic turn,” Lévi-Strauss’ resource to linguistics went beyond persuasive analogies. Contrariwise, his application of linguistic procedures strived to keep as strict a correspondence with his methodological sources of inspiration as possible. From his point of view, this is theoretically justifiable on the grounds that linguistic and phonological models not only encode the structure of language, but ultimately that of the human mind as well. Furthermore, as Lévi-Strauss himself has repeatedly argued, music, inasmuch as it can be considered a formal and relational system, also shares properties with the models of language proposed by structural linguistics. In fact, when outlining the main procedural rules for structural analysis, he drew extensively from analogies with music (Lévi-Strauss, 1955). Thus, for Lévi-Strauss music shares with language deep and revealing affinities, predicated - as we have already anticipated - on their both expressing the structure of the human mind.

Our bottom-up approach could be in a sense considered an inversion of the structuralist agenda: instead of translating music scores to linguistic-inspired formal models as part of a progressive approach to the structure of the mind, we built a symbolic logic to translate neuronal signals into music scores in order to be able to analyze their structure in search of potential homologies. Thus, our experiment in creating a music score from neuronal signals was intended to enable the comparison between music and raw neuronal activity, in similar terms to those outlined by structuralist analysis. Our results point to the same conclusion, showing the structures of music and neuronal activity to be homologous. Furthermore, the scores obtained were not only spontaneously tonal, but they also exhibited structure and features normally present in human-made musical creations. The original hypothesis of a parallelism between the neuronal code and the code of music holds, suggesting that some of the insights introduced by linguistic and semiotic theory might be a useful methodological resource to go beyond the limits set by metaphoric notions of “code.”

## Data availability statement

The raw data supporting the conclusions of this article will be made available by the authors, without undue reservation.

## Ethics statement

The animal study was reviewed and approved by the Local Ethics Committee (CEIB), Fleni Institute, Buenos Aires, Argentina.

## Author contributions

GN and DA contributed equally to the analysis of the data and elaboration of the manuscript. DA performed all the calculations. LM was in charge of the compositional analysis. All authors contributed to the article and approved the submitted version.

## Footnotes

1 “Homologous” within the context of the structuralist approach means that the codes in question share basic structural features, which in the case in point are expressed as complex mathematical properties. It does not imply strict commensurability or isomorphism (Lévi-Strauss, 1955).

2 Regarding this point, it should be taken into account that the term “structure” is deployed with different meanings in different disciplines, all of which converge and affect directly the work undertaken in the present paper: linguistics, anthropology, music and compositional analysis, fractal geometry and time series analysis. We use the term in its most general sense of a system of relationships.

3 The use of GPi neurons for this experiment is particularly suitable, since the GPi is a deep brain structure related not only to motor, but also to higher functions, such as associative and affective functions (Lanciego et al., 2012). The fact that the neuronal activity was recorded in control animals while awake excludes any potential effect of drugs in our results. Further experimental details can be found in Andres et al. (2014). The extrapolation of results to the human brain should always be made with caution, but mammalian models are the closest option for an invasive exploration of the healthy human brain.
